# Management of Anaesthesia and Cardiopulmonary Bypass in Paediatric Patients With Abdominal Tumours Invading the Inferior Vena Cava and Right Atrium: A Case Series of a Tertiary Children's Medical Centre in China

**DOI:** 10.1002/cnr2.70268

**Published:** 2025-06-29

**Authors:** Shangyingying Li, Hongzhen Xu, Jie Li, Ting Zhang, Jie Cui

**Affiliations:** ^1^ Department of Anaesthesiology Children's Hospital of Chongqing Medical University, National Clinical Research Center for Child Health and Disorders, Ministry of Education Key Laboratory of Child Development and Disorders Chongqing China; ^2^ Chongqing Key Laboratory of Child Neurodevelopment and Cognitive Disorders Chongqing China; ^3^ Department of Radiology Children's Hospital of Chongqing Medical University Chongqing China

**Keywords:** anaesthesia, CPB, IVC, right atrium, tumour thrombus

## Abstract

**Objectives:**

Paediatric patients with abdominal tumours associated with tumour thrombus in the inferior vena cava (IVC) and right atrium are relatively rare in clinical practice. Hence, we summarised the management strategies for anaesthesia and cardiopulmonary bypass (CPB) used during surgical treatment for these conditions through multidisciplinary cooperation.

**Methods:**

We collected the clinical data of paediatric patients who underwent surgery for tumour thrombus removal via CPB from January 2012 to December 2022 because their abdominal tumours had invaded the IVC and right atrium. We explored the strategies used to manage anaesthesia and CPB, assessed the incidence of intraoperative haemorrhage and arterial blood gas analysis, reported the incidence of blood transfusion and described the postoperative outcome and follow‐up.

**Results:**

A total of six paediatric patients underwent surgery under CPB to remove the tumour thrombus. Among them, two patients had nephroblastoma, one had renal clear cell carcinoma and three had hepatoblastoma. The average age of the six patients was 25.8 months. The average operation time was 459.8 min, and the average anaesthesia time was 553.1 min. The average CPB time was 150.3 min, and the average aortic block time was 46.1 min. The average hypothermic circulatory arrest time was 20 min. The average quantity infused was as follows: red blood concentrate (RBC): 5.1 units, cryoprecipitate: 3.2 units, fresh frozen plasma (FFP): 200 mL and platelets (PLTs): 4.2 units. The time of extubation ranged from 4 h to 8 days, and the average time spent in the intensive care unit (ICU) was 6.2 days after surgery. No serious complications occurred during the follow‐up period.

**Conclusions:**

The present retrospective study aims to share our clinical experience with the management strategies of anaesthesia and CPB. Steady induction of anaesthesia, intraoperative massive haemorrhage and critical intraoperative situations are the major challenges in anaesthesia management.

## 1 Introduction

Paediatric abdominal tumours simultaneously invading the inferior vena cava (IVC) and right atrium are relatively rare, but this disease is very serious. The incidence of Wilms tumours associated with IVC tumour thrombus is 4%–10%, and the incidence of its association with tumour invasion into the right atrium is less than 1% [1, 2]. Invasion of the IVC and right atrium also occurs in patients with other abdominal tumours, such as hepatoblastoma [3, 4, 5]. A recent retrospective study revealed that although all paediatric Wilms tumours with vascular extension received neoadjuvant chemotherapy, approximately 2% of patients had persistent right atrial thrombus extension and underwent cardiopulmonary bypass (CPB) [6]. Removing the tumour and the tumour thrombus under CPB is a difficult operation with a high risk of complications, and thoracic surgery combined with abdominal surgery is needed. Management strategies for anaesthesia and CPB for the surgical treatment of these complex conditions involving multidisciplinary cooperation have seldom been reported worldwide, especially for children.

Here, we summarise our clinical experience in the management strategy of anaesthesia and CPB for the removal of the tumour thrombus from the IVC and right atrium in six children. This information may provide a reference for similar complex operations in the future.

## 2 Materials and Methods

### 2.1 Patient Population

This study was approved by the Institutional Review Board of the Children's Hospital of Chongqing Medical University, Chongqing, China (approval number: 2022 [No. 416], approval date: 13 September 2022), and the waiver of informed consent was approved by the Medical Ethics Committee of Children's Hospital of Chongqing Medical University. The trial was registered before patient enrolment at clinicaltrials.gov (ChiCTR2200065847, link to the trial at the registration website: https://www.chictr.org.cn/showproj.html?proj=183005, Principal investigator: Jie Cui, Date of registration: 16 November 2022).

**FIGURE 1:**
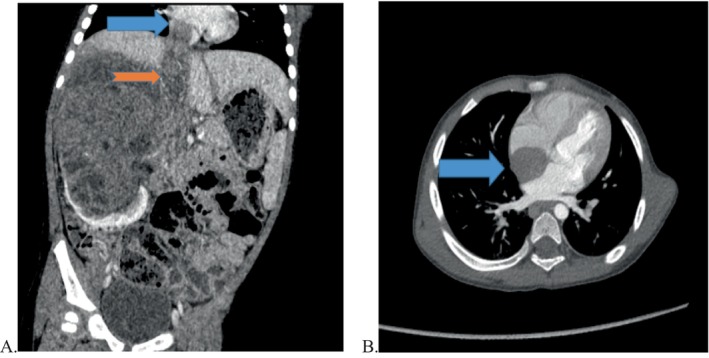
Preoperative computed tomography (CT) revealed a tumour thrombus in the inferior vena cava (IVC) (orange arrow in A) and a tumour thrombus in the right atrium (blue arrow in A and B).

**FIGURE 2:**
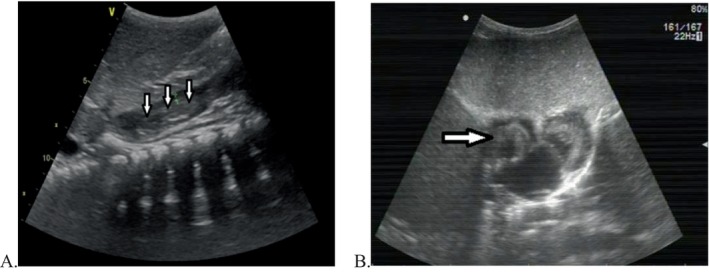
Preoperative transthoracic echocardiography (TTE) revealed a tumour thrombus in the inferior vena cava (IVC) (small arrows in A) and a tumour thrombus in the right atrium (large arrow in picture B).

We collected the clinical data of six paediatric patients who underwent surgery to remove a tumour thrombus under CPB from January 2012 to December 2022. The data were obtained from preoperative and postoperative medical records, preoperative and postoperative imaging data, surgical records, records of anaesthesia, records of CPB and other relevant records. The inclusion criteria were as follows: the primary abdominal tumour and the tumour thrombus in the IVC and right atrium were surgically excised under CPB. The exclusion criteria were as follows: no CPB, no right atrial thrombus extension, abnormal preoperative coagulation function and other types of surgery. Two patients were diagnosed with nephroblastoma, one with renal clear cell carcinoma and three with hepatoblastoma. The patients included three boys and three girls. The ages of the patients ranged from 14 to 41 months, and the results of preoperative imaging examinations, such as computed tomography (CT), revealed the size of the tumours, the size and position of the tumour thrombus, and so forth (Figure 1). Transthoracic echocardiography (TTE) revealed a tumour thrombus in the IVC and right atrium (Figure 2), and the tumour thrombus invaded the tricuspid valve in four patients before preoperative chemotherapy, three of whom had signs of right heart system obstruction. Preoperative chemotherapy was administered to all patients, and TTE confirmed that no tumour invaded the tricuspid valve after preoperative chemotherapy, as shown in Table 1. All of the patients underwent surgery to remove the abdominal tumour and the tumour thrombus in the IVC and right atrium under general anaesthesia and CPB. Among the patients with hepatoblastomas, two underwent hepatic lobectomies, and one underwent autologous liver transplantation.

**TABLE 1:** Clinical data and operative and postoperative characteristics of the six patients with abdominal tumours included in the study.

Item	Case 1	Case 2	Case 3	Case 4	Case 5	Case 6	Average (range)/range
Sex/age (months)	F/24	F/18	M/41	M/25	F/33	M/14	25.8 (14–41)
Weight (kg)	12.5	10	13.5	11.5	8.5	11	11.2 (8.5–13.5)
Primary malignancies	Wilm's tumour	Hepatoblastoma	Wilm's tumour	Hepatoblastoma	Hepatoblastoma	Renal clear cell carcinoma	
Size of tumour (mm) from CT	150 × 120 × 120	95 × 72 × 80	72 × 50 × 43	21.1 × 24.1 × 15.1	60.8 × 46.1 × 55.3	168 × 125 × 110	
TTE shows the thrombus invading the tricuspid valve before preoperative chemotherapy	No information	No	Yes	Yes	Yes	Yes	
Right heart system obstruction	No information	No	Pleural effusion	Pericardial effusion	No	Peritoneal effusion	
Preoperative chemotherapy (times)	4	3	2	5	4	3	4 (2–5)
CT shows the size of tumour thrombus in RA after chemotherapy (mm)	31 × 24 × 18	23.5 × 13.5 × 13	21 × 9	13.3 × 11.8 × 9.9	8.9 × 9.1	26 × 20 × 18	
TTE shows the thrombus invading the tricuspid valve after preoperative chemotherapy	No information	No	No	No	No	No	
Operative time (min)	445	580	495	365	464	410	459.8 (365–580)
Anaesthesia time (min)	510	745	565	450	559	490	553.1 (450–745)
Time of CPB (min)	150	261	151	115	134	91	150.3 (91–261)
Aortic clamping time (min)	45	23	68	53	38	49	46.1 (23–68)
Circulatory arrest time (min)	31	5	56	0	3	25	20 (0–56)
Cerebral perfusion time (min)	0	0	0	42	30	0	
Lowest temperature (°C)	22.8	20.5	23	28	24	26	24.1 (20.5–28)
MUF time (min)	Yes	Yes	Yes	Yes	Yes	Yes	
Blood loss (mL)	700	2800	200	40	100	2000	973.3 (40–2800)
Urinary volume (mL)	210	720	255	420	350	500	409.2 (210–720)
Second laparotomy to haemostasis	Yes	Yes	No	No	No	Yes	
RBC transfusion (U)	4.75	7.5	3	3	3	9.5	5.1 (3–9.5)
FFP (mL)	100	500	100	100	100	300	200 (100–500)
PLTs (U)	0	10	5	0	0	10	4.2 (0–10)
Cryoprecipitate (U)	0	8	2	2	3	4	3.2 (0–8)
Autologous blood transfusion	No	Yes	Yes	No	No	No	
TEE	No	Yes	No	Yes	Yes	Yes	
TEG	No	Yes	No	Yes	Yes	Yes	
Time to extubation after operation	14 h	8 days	19 h	16 h	4 h	13 h	4 h to 8 days
ICU stay after operation (days)	3	19	2	3	3	7	6.2 (2–19)
Postoperative chemotherapy (times)	9	2	15	4	7	1	6.3 (2–15)
Follow‐up	7 years, alive	5 years, alive	5 years, alive	2 years, alive	1 year, alive	6 months, alive	

Abbreviations: CPB, cardiopulmonary bypass; CT, computed tomography; FFP, fresh frozen plasma; ICU, intensive care unit; MUF, modified ultrafiltration; PLTs, platelets; RA, right atrium; RBC, red blood concentrate; TEE, transoesophageal echocardiography; TEG, thromboelastogram; TTE, transthoracic echocardiography.

### 2.2 Management and Follow‐Up

#### 2.2.1 Anaesthesia Procedure

All patients were subjected to ultrasound guidance for radial artery cannulation under sedation, and the invasive arterial pressure was monitored. Anaesthesia was subsequently induced with intravenous injection of 0.05 mg/kg midazolam, 2–3 mg/kg propofol, 1 μg/kg sufentanil, 0.6 mg/kg rocuronium, 0.1 mg/kg cisatracurium besilate and 0.01 mg/kg penehyclidine hydrochloride. The patients were intubated with a tracheal tube after 3–5 min and connected to the anaesthesia machine for ventilation. The PCV ~ VG model was employed for mechanical ventilation, with an oxygen concentration ranging from 35% to 40% in the air–oxygen mixture. The gas flow rate was set at 2 L/min, and the tidal volume ranged from 8 to 10 mL/kg. The respiratory rate was adjusted on the basis of the child's age, ensuring that the airway peak pressure remained below 30 cmH_2_O while maintaining PETCO_2_ levels within the range of 35–45 mmHg.

Ultrasound‐guided catheterisation of the right internal jugular vein and central venous pressure (CVP) monitoring were performed after endotracheal intubation. The invasive arterial blood pressure (IBP), heart rate (HR), percutaneous pulse oxygen saturation (SPO_2_), CVP, arterial blood gas analysis, thromboelastogram (TEG), nasopharyngeal temperature and urine volume were meticulously documented throughout the procedure. A transoesophageal echocardiography (TEE) probe was inserted to facilitate intraoperative monitoring. The intravenous administration of propofol at a rate of 4–6 mg/kg/h, sufentanil at a rate of 2–4 μg/kg/h, rocuronium at a rate of 1 mg/kg/h, or cisatracurium besilate at a rate of 1.5 mg/kg/h was initiated and sevoflurane inhalation at a concentration of 1%–2% was used for anaesthesia maintenance.

#### 2.2.2 Surgical Procedure and CPB

**FIGURE 3:**
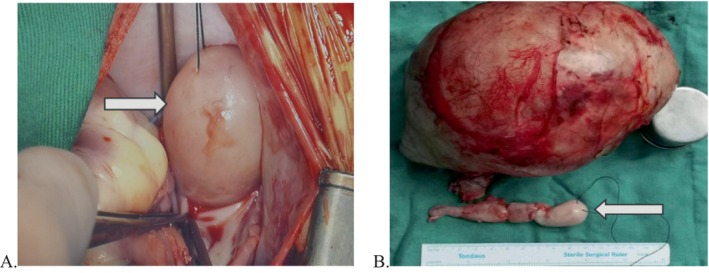
The intraoperative finding was the head of the tumour thrombus at the entrance of the right atrium (arrow in A). The excised abdominal tumour and tumour thrombus (arrow in B) are shown.

The primary tumour and the tumour thrombus in the IVC and right atrium were surgically excised from all paediatric patients (Figure 3). The abdominal incision was similar to that used for liver or kidney tumour resection. After abdominal tumour resection, median sternotomy and pericardiotomy were performed.

The abdominal cavity was filled with gauze following successful haemostasis in four children. Then, a simple suture was applied to close the incision, which was subsequently covered with a surgical film. The abdominal cavity of two patients was directly closed following thorough haemostasis. All of the patients underwent median sternotomy and pericardiotomy and were then administered heparin at a dosage of 3 mg/kg. The aorta and right atrium were cannulated to establish CPB for systemic cooling. The aortic root was occluded, and cold crystal myocardial protective solution was injected. The decision to utilise either deep hypothermic circulatory arrest (DHCA) or selective cerebral perfusion (SCP) was left to the discretion of the surgeon.

During CPB, intracardiac exploration and removal of the tumour thrombus were performed while the patient was gradually rewarmed. The patient's head was positioned in a lowered position to facilitate exhaust from the aorta before the aorta was opened, and the heartbeat resumed after the opening of the aorta. Upon reaching a core temperature of 37°C, the patients were gradually weaned off CPB until complete cessation of CPB flow was achieved. After the completion of modified ultrafiltration, protamine was used to neutralise the heparin, and the chest incision was closed as part of the standard surgical procedure. The abdominal cavity of four patients was reopened for assessment for intra‐abdominal haemorrhage, and the abdominal incision was subsequently closed following confirmation of its absence.

Arterial blood gas monitoring was performed to allow the anaesthesiologist to maintain patient stability during the operation procedure, and ultrafiltration was utilised to concentrate the blood and increase patient homeostasis. When the surgery finished, the patients were transferred to the intensive care unit (ICU) for close observation and further treatment.

#### 2.2.3 Postoperative Follow‐Up

All patients were regularly hospitalised for postoperative chemotherapy and followed up at our outpatient clinics. Generally, the patients were followed up once a month in the first year after surgery, once every 3 months in the second year, once every 6 months in the third year, and then once every 6 months or once a year thereafter. However, the follow‐up schedule may need to be adjusted according to the changes in the child's condition in some cases. Abdominal CT scans and echocardiography were performed for the children during the follow‐up visits. Survival information was collected from reports obtained during the follow‐up visits as well as through phone interviews with the patients' parents.

## 3 Results

A total of six paediatric patients with abdominal tumours invading the IVC and right atrium underwent surgical resection of the tumour and removal of the tumour thrombus under CPB. The primary malignancies included two cases of nephroblastoma, one case of renal clear cell carcinoma, and three cases of hepatoblastoma. In all the instances, complete surgical resection was successfully achieved for both the primary tumour and the tumour thrombus. The demographic characteristics, clinical data, and intraoperative details of all patients are presented in Table 1.

The mean age of all of these patients was 25.8 months (range: 14–41 months), with an equal distribution of three boys and three girls. The average operation time was 459.8 min (range: 365–580 min), whereas the mean anaesthesia time was 553.1 min (range: 450–745 min). The average duration of CPB was 150.3 min (range: 91–261 min). The average duration of aortic clamping was 46.1 min (range: 23–68 min). Hypothermic circulatory arrest was performed in five patients, for an average duration of 20 min (range: 0–56 min). One patient underwent SCP. The average quantity of red blood concentrate (RBC) transfused was 5.1 units (range: 3–9.5 units), that of cryoprecipitate was 3.2 units (range: 0–8 units), that of fresh frozen plasma (FFP) was 200 mL (range: 100–500 mL), and that of platelets (PLTs) was 4.2 units (range: 0–10 units). Four patients underwent TEG and TEE for intraoperative monitoring, whereas two patients received autologous blood transfusions during the procedure. The time of extubation ranged from 4 h to 8 days, and the average duration of stay in the ICU was 6.2 days (range: 2–19 days) after surgery. Postoperative chemotherapy was administered to all patients.

For five patients, the steps of the surgical procedure involved resection of the abdominal tumour and removal of the tumour thrombus in the IVC, followed by removal of the tumour thrombus in the right atrium under CPB. One patient underwent ex vivo hepatic resection and autologous liver transplantation under CPB to mitigate bleeding, followed by removal of the tumour thrombus from the IVC and right atrium.

**FIGURE 4:**
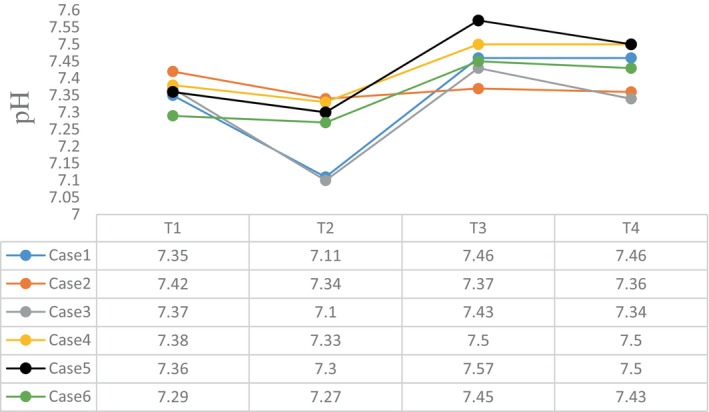
pH value at different time points. The abscissa axis represents different time points of surgery, and the vertical axis represents the value of pH. Different coloured lines represent different cases: T1: preoperative; T2: the lowest value at CPB; T3: at the end of CPB; T4: at the end of surgery. The standard pH value ranges from 7.35 to 7.45.

**FIGURE 5:**
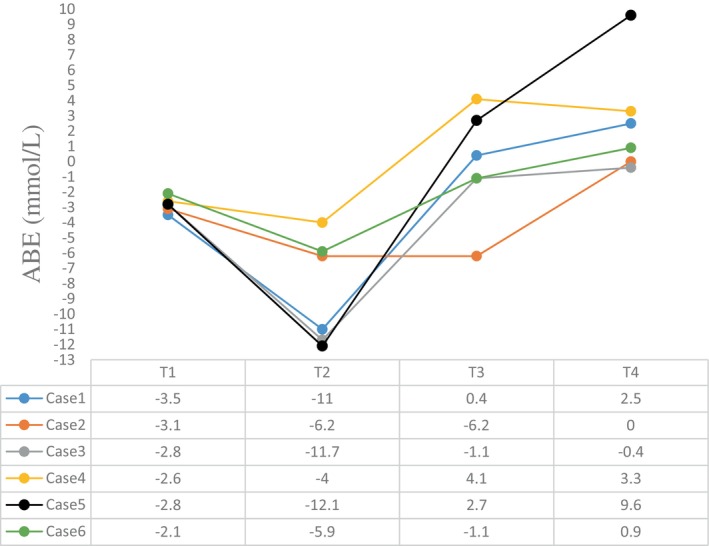
ABE levels at different time points. The abscissa axis represents different time points of surgery, and the vertical axis represents the value of ABE (mmol/L). Different coloured lines represent different cases: T1: preoperative; T2: the lowest value at CPB; T3: at the end of CPB; T4: at the end of surgery; ABE: actual base excess. The standard value of ABE ranges from −3 to 3 mmol/L.

**FIGURE 6:**
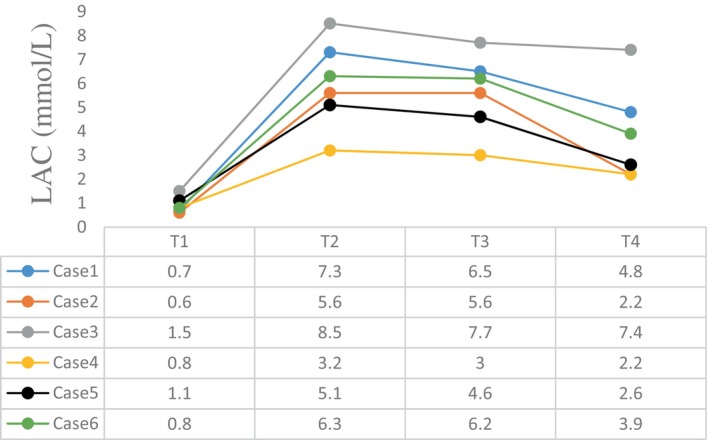
LAC levels at different time points. The abscissa axis represents different time points of surgery, and the vertical axis represents the value of LAC (mmol/L). Different coloured lines represent different cases: T1: preoperative; T2: lowest value at CPB; T3: at the end of CPB; T4: at the end of surgery; LAC: lactate. The standard value of LAC ranges from 0.7 to 2.1 mmol/L.

**FIGURE 7:**
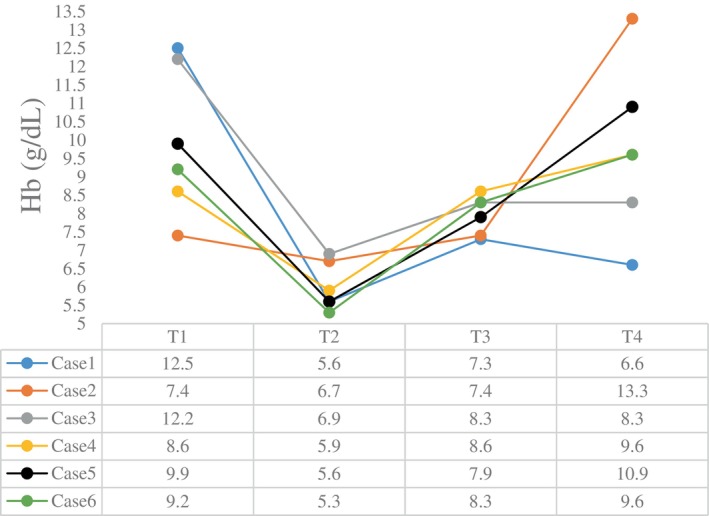
Hb levels at different time points. The abscissa axis represents different time points of surgery, and the vertical axis represents the value of Hb (g/dL). Different coloured lines represent different cases: T1: preoperative; T2: lowest value at CPB; T3: at the end of CPB; T4: at the end of surgery; Hb: haemoglobin. The standard value of Hb ranges from 11.0 to 15.0 g/dL.

The intraoperative blood gas monitoring results depicted in Figures 4, 5, 6, 7 reveal reduced pH values (standard value range: 7.35–7.45), indicative of metabolic acidosis; elevated levels of lactate (LAC) (standard value range: 0.7–2.1 mmol/L); and reduced haemoglobin (Hb) (standard value range of 110–150 g/L) during CPB, despite attempts at correction. The haemoglobin levels at the end of surgery were comparable to their preoperative values, whereas the pH, actual base excess (ABE) (standard value range: −3 to 3 mmol/L), and LAC values increased compared with their preoperative values.

The follow‐up period ranged from 6 months to 7 years, ending in December 2022. All patients survived without any occurrence of serious complications after surgery, such as tumour recurrence or tumour metastasis.

## 4 Discussion

It is highly challenging for surgeons to successfully excise primary tumours and tumour thrombi from paediatric abdominal tumours invading the IVC and right atrium. The mainstay of treatment involves multidisciplinary management, including thoracoabdominal surgery performed under CPB; however, perioperative anaesthesia management presents a major hurdle for anaesthetists. Nevertheless, there is limited literature available on the strategies employed in managing anaesthesia and CPB during this surgical procedure.

To ensure the perioperative safety of paediatric patients, anaesthesiologists should conduct comprehensive preoperative assessments and thorough preparations while also familiarising themselves with key aspects of intraoperative management for children, particularly those with right heart system obstruction, massive haemorrhage, and pulmonary embolism. Therefore, through the analysis of these six paediatric patients, we synthesised comprehensive strategies for effectively managing both anaesthesia and CPB during this intricate surgical procedure from various perspectives.

### 4.1 Preoperative Evaluation and Preparation

Adequate preoperative evaluation and preparation are crucial for surgically removing tumours invading the IVC and right atrium. All anaesthesiologists must engage in thorough communication with surgeons and perfusionists regarding the operative plan to develop safe and feasible anaesthesia and CPB strategies. The evaluation of the tumour thrombus location and extent, as well as its relationship with surrounding tissues from CT or ultrasonic reports, is crucial. The primary focus should be on evaluating the presence of clinical manifestations indicative of right heart system obstruction, such as shortness of breath, dyspnoea, positional discomfort and haemodynamic instability.

In this study, preoperative CT and cardiac ultrasound revealed tricuspid valve invasion by the tumour thrombus in four paediatric patients, accompanied by pleural effusion, peritoneal effusion or pericardial effusion in three patients before the initiation of chemotherapy. After several months of neoadjuvant chemotherapy, a significant reduction in the size of the right atrial tumour thrombus was observed. The size of the tumour thrombus in the right atrium ranged from 8.9 × 9.1 mm to 31 × 24 × 18 mm before surgery. None of the children presented with any clinical manifestations of right heart system obstruction before surgery.

### 4.2 Anaesthesia Induction

The induction of anaesthesia in these patients poses a significant procedural risk owing to the potential for right heart system obstruction and pulmonary embolism. Therefore, before anaesthesia induction, debridement, adrenaline and atropine are administered as emergency measures. In addition, all of the children included in this study underwent sedation and invasive blood pressure monitoring through ultrasound‐guided arterial puncture. The next step involved the administration of intravenous anaesthesia and endotracheal intubation. In cases where hypotension occurs following induction, prompt management with rehydration and/or vasoconstrictor drug infusion is recommended. The children underwent ultrasound‐guided internal jugular vein catheterisation following endotracheal intubation; however, caution was exercised to prevent excessive advancement of the guidewire to avoid contact with the embolus, thereby ensuring accurate wire placement under ultrasonic guidance.

Both the surgeon and perfusionist are required during anaesthesia induction, ensuring the immediate establishment of CPB in case of an emergency. If the preoperative evaluation reveals close adherence of the tumour thrombus to the vessel wall, it becomes imperative to establish more appropriate venous access for facilitating fluid transfusion in patients with significant haemorrhage. If a child presents with clinical manifestations of right heart system obstruction before surgery, the exacerbation of right heart flow obstruction and subsequent irreversible hypoxia due to the effects of anaesthesia induction can be mitigated by placing the patient in the Trendelenburg position to enhance venous return while simultaneously administering rehydration and vasoconstrictor drugs to ameliorate symptoms. The use of positive inotropic drugs should be avoided because they may exacerbate the obstruction. If the aforementioned treatment fails to resolve the obstruction or leads to a decrease in HR, arrhythmia or even cardiac arrest, immediate thoracic cavity opening is recommended for establishing CPB to ensure the safety of the child.

A TEE probe can be inserted following the administration of anaesthesia. TEE not only confirms the presence of a tumour thrombus but also assesses its exact location and size before surgery, aiding surgeons in formulating an intraoperative protocol and diagnosing pulmonary embolism. Moreover, TEE also confirms the absence of thrombi after surgery and allows monitoring of cardiac function and blood volume [7, 8, 9]. TEE was utilised for monitoring four patients in our study. Unfortunately, the TEE findings were not recorded.

### 4.3 Management of Anaesthesia

#### 4.3.1 Management of Massive Bleeding

The management of anaesthesia poses significant challenges due to the substantial amount of intraoperative blood loss and associated complications. Among the five patients, the initial step in the surgical procedure involved resection of the abdominal tumours and removal of the tumour thrombus from the IVC, followed by subsequent removal of the tumour thrombus from the right atrium under CPB. Following tumour resection, occlusion was applied to both the renal/hepatic veins and the IVC side of the tumour, after which the IVC was incised for complete removal of the tumour thrombus. Making surgical incisions into the IVC often leads to rapid and substantial haemorrhaging. Anaesthesiologists must promptly and accurately assess blood loss and the bleeding rate in paediatric patients because of their limited blood volume, necessitating timely blood transfusion.

In addition, the infusion of vasoactive drugs during the procedure is essential for maintaining adequate perfusion pressure in vital organs. In particular, the tumour thrombus may tightly adhere to the wall of the IVC, and separation of the tumour thrombus can lead to significant haemorrhage. Surgeons should be reminded about ensuring haemostasis of the abdominal wound and closely monitoring abdominal drainage during CPB, given the impact of heparin on systemic coagulation function.

Abdominal tumour wounds are highly susceptible to rebleeding, which can be difficult to detect. In our study, the abdominal cavity was closed directly in two patients who underwent a second laparotomy for haemostasis and massive transfusion due to persistent hypovolaemia and hypochromes after CPB. Therefore, we recommend the use of gauze to fill the abdominal cavity after abdominal surgery, followed by closure of the incision using a simple suture technique and covering the incision with a surgical film to facilitate subsequent reopening for assessment of abdominal bleeding after the reversal of heparinisation.

In our study, routine closure of the abdominal cavity was performed in four patients after the absence of bleeding had been confirmed. After the surgeon examined the abdominal cavity following CPB, massive bleeding was detected in one patient. Prompt measures were taken to achieve haemostasis, and blood transfusions were administered to prevent potential life‐threatening complications associated with persistent postoperative bleeding.

#### 4.3.2 Autologous Blood Transfusion

The efficacy of autologous blood transfusion in reducing the need for allogeneic blood transfusions in patients with massive bleeding has been well established; however, the optimal utilisation of intraoperative autologous blood transfusion in patients with tumours has not been determined. Recent studies have indicated that intraoperative autologous blood transfusion is not associated with adverse oncologic outcomes and is considered a safe procedure [10, 11, 12]. In this study, two children underwent intraoperative autologous blood transfusion without experiencing any new distant metastasis or oncologic complications after the operation.

#### 4.3.3 Management of Embolism

The fatal complication is embolism. When the tumour embolises at the subhepatic IVC, the tumour embolus may dislodge and migrate into the right atrium, potentially resulting in pulmonary embolism. This occurrence is exceptionally uncommon, but its mortality rate in clinical settings is exceedingly high. The immediate establishment of CPB is imperative once a pulmonary embolism arises.

A study reported that a 68‐year‐old female patient developed a pulmonary embolism during dissection of the IVC for resection of a pararenal tumour extending into the retrohepatic IVC. Emergency CPB was established to remove the tumour embolism in the pulmonary artery, and the patient successfully recovered after surgery [13]. Therefore, while removing abdominal tumour thrombi, anaesthetists should maintain vigilant monitoring for pulmonary embolism indicators, including abrupt reductions in end‐expiration carbon dioxide waveforms, diminished oxygen saturation levels, arterial hypotension, tachycardia and elevated CVP, in paediatric patients. The surgeon should be promptly notified to halt ongoing abdominal surgery. If necessary, the cardiac surgeon should initiate CPB.

Another rare complication is the occurrence of air embolism. Surgical damage to the IVC can result in air embolism [14], as evidenced by a reported case in France involving a 4‐year‐old child who experienced a significant intraoperative incision in the IVC, ultimately leading to fatality due to air embolism [15]. Fortunately, none of the six patients in this study experienced any significant complications.

### 4.4 Management of CPB

#### 4.4.1 Strategy for the CPB

Whether CPB is necessary for the treatment of abdominal tumours with IVC tumour thrombus primarily depends on the degree of invasion by the thrombus into the IVC or the heart. In cases where the tumour thrombus is small, has not invaded the heart, and is not significantly adherent to the IVC, surgeons often opt for transient occlusion of the proximal end of the IVC to prevent thrombus migration, which could lead to right ventricular outflow tract obstruction and pulmonary embolism. Simultaneously, occlusion of the distal end of the IVC is performed to prevent massive haemorrhage. A rapid incision is subsequently made in the IVC to extract the thrombus, followed by repair of the IVC incision, thereby completing the procedure [16].

However, when the tumour thrombus invades the heart or exhibits extensive adherence to the IVC, two major risks emerge that significantly increase the surgical complexity and risk of life‐threatening consequences. The first is the potential for pulmonary embolism caused by thrombus migration. Second, the greatest challenges and dangers during such surgeries are cardiac or IVC rupture during the process of detaching the thrombus, with the associated risks of catastrophic haemorrhage and air embolism stemming from the crevasse.

Research has reported the case of a patient with renal cell carcinoma who underwent removal of a tumour thrombus from the IVC and pulmonary artery. During the removal of the IVC thrombus, vascular rupture occurred, with a bleeding volume of 22 000 mL. Although patients survive, this type of massive blood loss can be fatal [17].

CPB, through cardiac arrest and hypothermia, not only mitigates the risk of pulmonary embolism, safeguarding organ functions but also, by rapidly reinfusing the surgical field, avoids catastrophic haemorrhage. This not only ensures patient safety but also allows surgeons to perform tumour thrombus removal in a bloodless surgical field with a static heart, providing favourable conditions for thorough thrombus removal. In this study, all six paediatric patients presented with tumour thrombi that had invaded the heart or were extensively and tightly adhered to the IVC. To ensure patient safety and achieve complete thrombus removal, CPB was employed for thrombus removal in all patients. This is consistent with the method reported by Zacek et al. [18].

During the surgeries reported here, no massive blood transfusions were required due to haemorrhage. However, two patients required significant blood product transfusions due to profuse haemorrhage from the abdominal tumour bed after CPB was discontinued. Notably, none of the patients experienced critical events, such as pulmonary embolism or right ventricular outflow tract obstruction. Consequently, we recommend CPB for cases involving tight adhesion of the tumour thrombus to the vascular wall, thrombus extension into the heart, high risks of massive haemorrhage and pulmonary embolism or prolonged IVC occlusion time. For patients with relatively intact tumours and thrombi without an intra‐atrial component, we advise having CPB on standby in cases of pulmonary embolism or massive haemorrhage.

#### 4.4.2 DHCA

The choice of the CPB technique depends primarily on the complexity of the surgery and the required duration of circulatory arrest. In this study, six paediatric patients had tumour thrombi invading the IVC and right atrium, rendering IVC cannulation infeasible. Therefore, we performed cannulation of the right atrium and aorta to establish CPB for cooling. There are two primary techniques for CPB during this type of surgery: DHCA and SCP [18]. Both techniques are capable of reducing brain metabolism and protecting brain function, contributing to a bloodless surgical field, optimal visualisation of the tumour thrombus, and complete removal of the thrombus by the surgeon, thereby reducing the risk of complications and intraoperative adverse events. During DHCA, the temperature is typically adjusted to an appropriate level on the basis of the duration of circulatory arrest. When the temperature ranges from 28°C to 18°C, the safe duration of circulatory arrest is between 15 and 45 min [19]. However, DHCA undoubtedly prolongs the duration of the procedure and negatively affects bleeding and the inflammatory response. When the duration of circulatory arrest exceeds the anticipated duration, the incidence of neurological complications increases significantly. In this study, we chose the SCP as the initial procedure for five children. In three cases, after opening the right atrium, the surgeon reported that the tumour thrombus was poorly exposed and difficult to remove completely. Thus, the surgeon requested performing DHCA and simultaneously removing the arteriovenous cannula to provide a clearer surgical field. Multiple studies have demonstrated that the SCP provides superior neuroprotection to brain function [18, 20, 21]. Furthermore, the duration of cooling and rewarming after SCP is shorter than that after DHCA, effectively reducing the time required for CPB [22]. Therefore, we recommend prioritising SCP in such surgeries [23]. However, for paediatric patients with poor surgical exposure and complex intraoperative manipulations, surgeons may prefer to remove arteriovenous cannulas under DHCA to provide a clearer surgical field and facilitate the operation, thereby shortening the operative time and improving surgical success rates. Ultimately, the choice of extracorporeal circulation modality should be decided in consultation with the surgeon.

#### 4.4.3 SCP

SCPs are classified primarily on the basis of the direction of perfusion: antegrade cerebral perfusion (ACP) and retrograde cerebral perfusion (RCP). Both techniques effectively provide continuous oxygen and nutrients to the brain, contributing to minimising brain injury during surgical procedures. The choice between ACP and RCP in practical applications depends on various factors, including the type of surgery, the patient's condition, and the surgeon's experience. Studies have shown that RCP may be more suitable for surgeries requiring prolonged aortic occlusion [24]. However, ACP is still used as a routine procedure in clinical practice, possibly because ACP is more in line with human physiological processes [25]. Therefore, RCP is commonly used for patients who cannot undergo SCP or as an emergency measure to remove an intracranial air embolism [26]. In this study, considering the complex condition of paediatric patients, the potential for extended cerebral perfusion time, and the lack of consensus on RCP in paediatric flows, ACP was deemed more appropriate.

ACP is also distinguished by the location of perfusion: unilateral cerebral perfusion (UCP) and bilateral cerebral perfusion (BCP). The selection between the UCP and BCP is primarily determined by the integrity of the circle of Willis and the adequacy of collateral circulation in the patient's brain. Although BCP, achieved through cannulation of the right axillary artery and left common carotid artery, ensures adequate blood supply to both cerebral hemispheres, it is technically more complex and may lead to inadequate perfusion, pressure instability and additional risks. Moreover, the delicate vasculature in children increases the risk of vascular injury from peripheral artery cannulation. Research has indicated that, during hypothermic CPB, UCP and RCP exhibit no significant difference in their impact on postoperative neurological function in paediatric patients. This may be attributed to the abundant collateral vessels in the brain and the utilisation of neurological monitoring tools such as cerebral oxygenation and electroencephalography.

Both methods (DHCA and SCP) can lead to the development of ischaemia and hypoxia, resulting in a significant increase in LAC levels, as observed in the six patients. Therefore, we recommend ultrafiltration as a routine procedure to optimise patient homeostasis, concentrate blood components and alleviate inflammatory responses. In this study, the physiology of all patients was basically within the normal range, with a decrease in LAC and an increase in haemoglobin content at the end of CPB. No significant brain dysfunction was observed during postoperative follow‐up, and all patients recovered satisfactorily.

### 4.5 Management of Coagulation Function

Maintaining coagulation function in patients undergoing such surgery is challenging. Massive blood loss, heparin anticoagulation, the dilution and disruption of coagulation factors and PLTs by CPB, and the inhibition of coagulation function at low temperatures were all factors that affected coagulation function in our study. Massive blood loss leads to the loss of coagulation components, and the amount of bleeding ranged from 402 800 mL in these six patients. The anticoagulant effect of heparin is inevitable because of the establishment of CPB. Thus, coagulation function is abnormal in patients during CPB, and they are more prone to bleeding. Adequate dosing with protamine sulphate is used to reverse the anticoagulant effect of heparin at the end of the CPB. Coagulopathy after CPB is common and leads to excessive bleeding, transfusions and worse outcomes [27]. The dilution of coagulation factors and PLTs is a consequence of the use of CPB [28]. TEG was performed after the end of CPB, and the results were used to guide the decision to transfuse the PPF, PLT or cryoprecipitate. In our study, PPF was infused into all of the patients, PLT to three and cryoprecipitate to five. The effects of hypothermia on coagulation may represent a two‐edged sword. On the one hand, the inhibition of coagulation can have positive effects on improving the microcirculation; on the other hand, this could lead to an increased risk of bleeding, especially if they are actively bleeding [29]. Hypothermia causes PLT dysfunction and reduced coagulation factor activity (significantly below 33°C), and PLT dysfunction and impaired enzymatic activity of coagulation factors are reversible with normalisation of body temperature [28]. Therefore, before the end of CPB, we ensured that the core temperature of patients reached 37°C in our study.

### 4.6 Management of a Special Case (Case 2)

In this study, despite multiple rounds of preoperative chemotherapy resulting in a decrease in tumour volume in one patient with hepatoblastoma, extensive invasion was still observed in liver segments IV, V and VIII as well as in the IVC and right atrium. Owing to the limitations of conventional approaches in achieving complete tumour removal and the significantly high risk of massive bleeding, ex vivo hepatic resection combined with autologous liver transplantation under extracorporeal circulation was chosen as the surgical strategy for removing the tumour thrombus from the IVC and right atrium. CPB was established via intubation through the distal end of the subhepatic IVC, right atrium and ascending aorta. The utilisation of CPB effectively mitigated the occurrence of IVC and portal vein obstruction and internal organ congestion resulting from prolonged surgical procedures during conventional semi‐isolated hepatectomy. Moreover, this approach decreases the risk of massive intraoperative bleeding. Although the duration of anaesthesia, operation and CPB was longer in this particular child than in the other five children, they recovered well and were discharged from the hospital after 3 weeks. A favourable prognosis was observed after a 5‐year follow‐up period.

### 4.7 Body Temperature Management

The wide incision and infusion of large volumes of fluid and blood during such surgery pose significant challenges in maintaining body temperature. However, the use of the CPB effectively manages the temperature [30], ensuring a stable temperature before CPB downtime. However, the risk of hypothermia persists in some patients after the cessation of CPB. Multiple methods are used to maintain body temperature in patients with hypothermia after CPB, including the use of a water blanket, warm air heater and infusion heater. Hypothermia can affect blood coagulation function and may affect the internal environment and prognosis of paediatric patients.

Some limitations of this retrospective study should be considered. First, the number of included cases was too small, with six patients. We did not analyse the relevant factors with statistical methods or statistical tests and only provided a description of the data. Second, some information was incomplete. For example, we did not provide the results of echocardiography before or after preoperative chemotherapy for Patient 1. We also did not provide TEE findings during the monitoring process because they were not recorded. Third, the operations of the six patients were performed by different surgeons, and the anaesthesia was managed by different anaesthesiologists.

## 5 Conclusions

The removal of tumour thrombi under CPB is a highly effective treatment for paediatric patients with abdominal tumours invading the IVC and right atrium. Adequate preoperative evaluation and preparation are essential for successful anaesthesia management. Moreover, we strongly recommend implementing close intraoperative monitoring of such patients, ensuring effective communication with surgeons and perfusionists and establishing a scheme for managing critical intraoperative situations to ensure the safety of anaesthesia induction. There are few clinically relevant reports on anaesthesia and CPB management in such paediatric patients; therefore, this paper mainly describes our clinical experience in managing anaesthesia for the surgical removal of tumour thrombi from the IVC and right atrium under CPB.

## Author Contributions

**Shangyingying Li:** conceptualization (equal), data curation (equal), investigation (equal), resources (equal), writing – original draft (lead). **Hongzhen Xu:** conceptualization (equal), investigation (equal), supervision (equal). **Jie Li:** investigation (equal), methodology (equal), supervision (equal), writing – review and editing (equal). **Ting Zhang:** investigation (equal), resources (equal), visualization (equal). **Jie Cui:** conceptualization (equal), funding acquisition (equal), investigation (equal), methodology (equal), project administration (equal), validation (equal), writing – review and editing (lead).

## Ethics Statement

All methods were applied in accordance with the relevant guidelines and regulations, and ethical approval for this study (Ethical Committee 2022 [No. 416]) was provided by the Institutional Review Board of the Children's Hospital Affiliated with Chongqing Medical University, Chongqing, China (Chairperson Professor Lu Zhongyi), on 13 September 2022. This study was registered at chictr.org.cn (ChiCTR2200065847, Principal investigator: Jie Cui, Date of registration: 16 November 2022) before its implementation.

## Consent

The authors have nothing to report.

## Conflicts of Interest

The authors declare no conflicts of interest.

## Data Availability

All the data generated or analysed during this study are included in this published article.
